# Supplementary figures and images for: Costing the Scale-Up of a National Primary School-Based Fluoride Varnish Program for Aboriginal Children Using Dental Assistants in Australia
Source: Int J Environ Res Public Health. 2020 Nov 26;17(23):8774. doi: 10.3390/ijerph17238774 (PMC7730616; doi:10.3390/ijerph17238774)

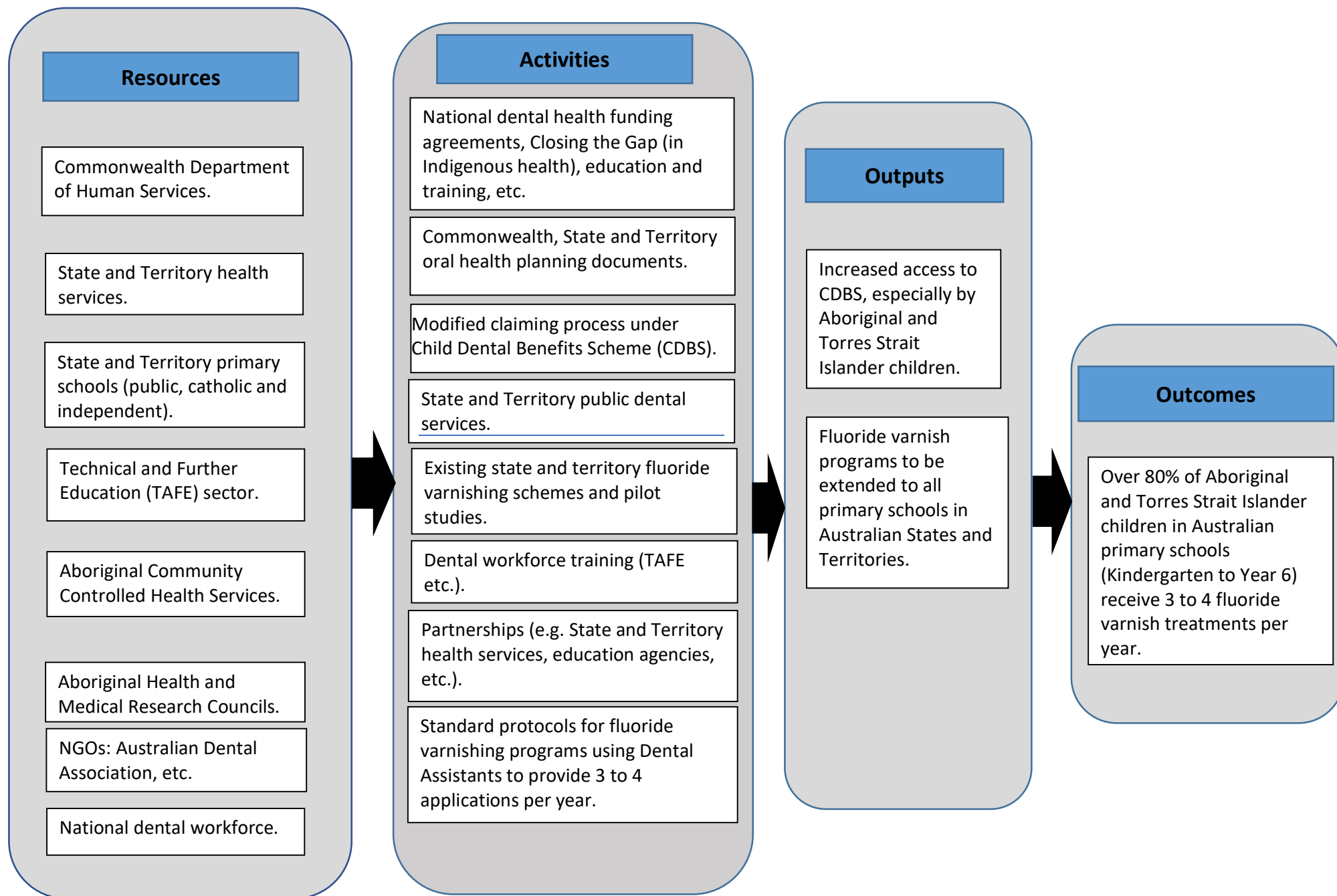

Supplement: Supplementary file 1 [file ijerph-17-08774-s001.pdf]
